# The evolving value assessment of cancer therapies: Results from a modified Delphi study

**DOI:** 10.1016/j.hpopen.2024.100116

**Published:** 2024-03-01

**Authors:** Myrto Lee, Hugo Larose, Martin Gräbeldinger, Jon Williams, Anne-Marie Baird, Susan Brown, Johannes Bruns, Russell Clark, Javier Cortes, Giuseppe Curigliano, Andrea Ferris, Louis P. Garrison, Y.K. Gupta, Ravindran Kanesvaran, Gary Lyman, Luca Pani, Zack Pemberton-Whiteley, Tomas Salmonson, Peter Sawicki, Barry Stein, Dong-Churl Suh, Galina Velikova, Jens Grueger

**Affiliations:** a Boston Consulting Group, London, UK; b Boston Consulting Group, Paris, France; c Lung Cancer Europe, Switzerland; d Susan G. Komen Foundation, USA; e Deutsche Krebsgesellschaft, Germany; f Cancer Technology Applications; Spesana, Inc, USA; g International Breast Cancer Center, Spain; h Department of Oncology and Hemato-Oncology, University of Milan, Division of Early Drug Development, European Institute of Oncology, IRCCS, Italy; i LUNGevity, USA; j University of Washington, USA; k All India Institute of Medical Science Bhopal, India; l Division of Medical Oncology, National Cancer Centre Singapore, Singapore; m Fred Hutchinson Cancer Research Center, USA; n University of Miami, Università di Modena e Reggio Emilia, Italy; o Leukaemia Care, UK, Acute Leukemia Advocates Network (ALAN), Switzerland, Blood Cancer Alliance (BCA), UK; p Consilium Salmonson & Hemmings, Belgium; q University of Cologne, Germany; r Cancer Colorectal Canada, Canada; s Chung-Ang University, South Korea; Rutgers, The State University of New Jersey, USA; t University of Leeds, UK; u Boston Consulting Group, Switzerland, Zurich, University of Washington, DC, USA

## Abstract

The move toward early detection and treatment of cancer presents challenges for value assessment using traditional endpoints. Current cancer management rarely considers the full economic and societal benefits of therapies. Our study used a modified Delphi process to develop principles for defining and assessing value of cancer therapies that aligns with the current trajectory of oncology research and reflects broader notions of value. 24 experts participated in consensus-building activities across 5 months (16 took part in structured interactions, including a survey, plenary sessions, interviews, and off-line discussions, while 8 participated in interviews). Discussion focused on: 1) which oncology-relevant endpoints should be used for assessing treatments for early-stage cancer and access decisions for early-stage treatments, and 2) the importance of additional value components and how these can be integrated in value assessments. The expert group reached consensus on 4 principles in relation to the first area (consider oncology-relevant endpoints other than overall survival; build evidence for endpoints that provide earlier indication of efficacy; develop evidence for the next generation of predictive measures; use managed entry agreements supported by ongoing evidence collection to address decision-maker evidence needs) and 3 principles in relation to the second (routinely use patient reported outcomes in value assessments; assess broad economic impact of new medicines; consider other value aspects of relevance to patients and society).

## Highlights

• Value assessment frameworks help guide decisions in a standardized and transparent fashion.
• Particularly in early-stage cancers, payers balance early access with maturity of data.
• We propose principles for defining and assessing the value of cancer therapies.
• The principles can facilitate discussion and decision-making on treatment development and evaluation.
• Next steps could include evolving existing value frameworks, to improve cancer outcomes.

## 1 Introduction

Value assessments play a key role in determining access to cancer treatments. To optimize outcomes and enable the most effective allocation of limited payer resources, clinical benefits must be balanced against the costs and potential risks of a therapy. Concepts of value are not static, however, and frameworks for assessing value need to evolve in parallel with scientific advances if they are to remain fit for purpose.

Rapid developments in cancer research, combined with a fundamental shift toward earlier diagnoses and earlier, more personalized, treatments, have improved outcomes for people with cancer around the world [1], [2], [3], [4], [5], [6]. Oncology science continues to deliver advancements that can lead to more targeted and effective treatments. However, if patients are to benefit fully from these developments, a shift in approach to value assessment is required to improve access to innovative therapies.

**Fig. 1:**
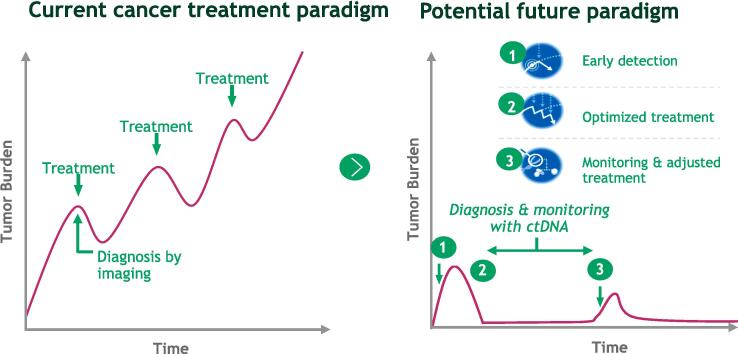
Illustrative paradigm shift in cancer treatment with curative intent.

Innovative treatments are particularly important for maximizing the potential benefits of early detection. The current cancer treatment paradigm generally aims for remission, followed by administering the next round of treatments on relapse (see Fig. 1). In a future paradigm based on early detection, by contrast, optimal treatment would often begin with monitoring and assessment to inform personalized treatments that are more localized and targeted, with curative intent [7]. To this end, the identification and utilization of additional oncology-relevant measures should be considered in terms of their role in accelerating the detection of even nascent cancers, speeding up drug development, and better informing treatment pathways and value assessments.

Funding decisions can be extremely complex because the cost of a new therapy does not always offset other healthcare expenses. Payers and health technology assessment bodies (HTA) account for the impact of medicines on health systems on aspects beyond the immediate cost to varying extents; some (e.g., the U.S.’s Institute for Clinical and Economic Review (ICER)) may include quantitative assessment of societal impacts [8], [9]. Further complexities arise when assessing the value of new treatments that show positive therapeutic effects without fully mature clinical data, as the potential value of earlier access must be weighed against safety or efficacy uncertainties. Regulators would have already made such deliberations before payers use the same clinical data but accompanied with cost data to make reimbursement decisions.

Overall Survival (OS) remains the standard endpoint in many contexts for payer decision-making regarding the clinical value of new oncology medicines. While OS is undeniably important, its usage can limit access to new medicines that could improve treatment results, especially when assessing new therapies for early-stage disease. OS data may be difficult to collect, could take many years to mature, and/or could be confounded by subsequent therapies [10], [11], [12]. Waiting sufficiently long to have mature OS data to make access decisions can mean that people with cancer today will not have the option of a treatment that could make them eligible for additional future treatments. Other oncology-relevant endpoints may thus be more appropriate for assessing treatments for early-stage cancers (see Supplemental Table 1).

Ultimately, it is important that people with cancer receive medicines as early as possible where there is sufficient evidence of efficacy and understanding of the treatment’s safety profile/tolerability. To achieve this goal, sustained collaboration among manufacturers, regulators, and payers is needed to define the value of oncology medicines in terms of clinical and other value components, including economic benefit, broader benefits for society at large, and value from the perspective of people with cancer (see Supplemental Table 2).

Value assessment in oncology has been recently reviewed [13], and in this study we build on that body of work by convening experts from a varied stakeholder-group to develop consensus across countries and stakeholder-groups to key outstanding questions. We also believe that our study is broader in scope, with considerations beyond endpoints. For this purpose, we employed a modified Delphi process, in which multidisciplinary experts were iteratively polled and convened for discussion to achieve consensus, on principles for defining and assessing the value of cancer therapies in a manner that both aligns with the current trajectory of oncology research and reflects broader notions of value. In particular, we sought to answer 2 questions:

1) Which oncology-relevant endpoints should be used to assess the benefit of treatments for early-stage cancer in clinical trials, and access decisions for early-stage cancer treatments?
2) Which additional value components are important in oncology and how can they be integrated in value assessments within healthcare systems and processes?

## 2 Materials and methods

**Fig. 2:**
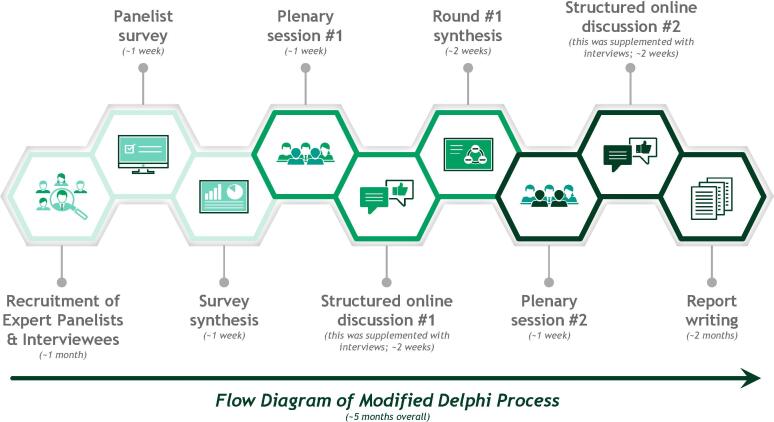
Flow diagram of modified Delphi process.

For this study we organised a modified Delphi process, which is a method for developing expert consensus on challenging topics through iterated rounds of polling and discussion (see Fig. 2), widely used in areas of healthcare where scientific evidence is insufficient or contradictory [14]. The goal of this process was to stimulate dialogue around appropriate value assessment, with a focus on early-stage disease, and ultimately reach consensus around a set of easily understandable, broadly applicable, and actionable principles for defining and assessing the value of cancer therapies.

### 2.1 Expert recruitment

The study team compiled a list of experts from the global cancer care ecosystem using purposive sampling. 16 expert panelists and 8 expert interviewees completed the exercise (see supplemental methods), including patient advocates, oncologists, health economists, regulators, members of payer and HTA bodies, and representatives from professional societies. The group included experts in a diverse range of cancer types as well as perspectives from 14 countries (Australia, Canada, China, Germany, India, Ireland, Italy, Japan, Singapore, South Korea, Spain, Sweden, the U.K., and the U.S.). Only experts who did not currently work at regulatory agencies, payers, or HTA bodies were invited to participate to protect against potential conflicts of interest. Some experts were provided compensation for their time in participating, and some experts chose to forego compensation (note, none of the experts were employed by a pharmaceutical company at the time of this project). All participants were briefed ahead of time on what to expect and consented to the process and the use that would be made of their contributions.

### 2.2 Consensus-Building process

Over a period of 5 months, 16 members of the expert group participated in 2 rounds of structured interactions. The first round began with members completing quantitative and qualitative items on a survey (see supplemental methods) to determine their current views on 4 dimensions of value (Clinical benefit; Benefit to patients and/or caregivers; Benefit to the healthcare system; Social / macro benefit). Expert panelists were also asked by the study team of independent consultants for their views on the suitability of 12 possible clinical endpoints for use in cancer trials (see Supplemental Table 1); the study team then summarised these views ahead of the first discussion.

Each round of interactions comprised a pre-read that included the results from the survey that preceded each plenary and agenda and objectives for the discussion. These were circulated 7 days before the meeting and a virtual plenary meeting (panel) followed by online discussions. Plenary sessions took the form of a 4-hour workshop-style meeting, with breakout sessions in which groups of experts discussed specific cancers. Expert panelists were encouraged to find common ground and to explore the reasons for differing views. Structured discussions then continued online on the secure social platform Within3.

In parallel with the plenary sessions and online discussions, the study team conducted structured interviews via videoconference with each expert panelist, and with 8 additional expert interviewees, using an interview guide developed with reference to initial survey results. These interviews provided additional qualitative information to assist in understanding the experts’ views and allowed the study team to clarify points of uncertainty. Interviews were also used to gather contributions from expert panelists who were unable to join one of the plenary sessions.

The study team conducted multiple rounds of polling during the second set of online discussions to determine where consensus was building and iteratively refined the principles to reduce or eliminate disagreements until expert panelists reached agreement. Final consensus on the wording of principles was established by co-authoring a consensus report. The study team prepared an initial draft presenting their understanding of the consensus position. The expert panelists and interviewees then offered revisions.

## 3 Results

### 3.1 Principles on oncology-relevant endpoints

The group identified four consensus principles pertaining to oncology-relevant endpoints for consideration in value assessments, particularly for early-stage cancers.

#### 3.1.1 Principle 1 Consider oncology-relevant endpoints other than OS which have intrinsic value for decision making

In early-stage cancer, OS data takes time to mature or may not be possible to collect in the longer term in early-stage disease. Indication, intent of treatment, and feasibility of measuring patient-relevant outcomes (e.g., event-free survival (EFS), disease-free survival (DFS), relapse-free survival (RFS)) within a reasonable timeframe should be evaluated when considering oncology-relevant endpoints as alternative to OS in value assessments. See Supplemental Table 1 for a decision of these and other oncology-relevant endpoints.

Access and funding decisions for cancer treatments currently focus primarily on OS data to determine treatment efficacy [15]. However, it can take years to collect the data, especially for early-stage disease [10], [16], and participants in clinical trials often start other treatments during follow up, confounding OS data for the initial therapy [11], [12]. People with cancer also may not survive long enough, or may have disease that is too advanced, to benefit from access to new treatments while data matures.

**Fig. 3:**
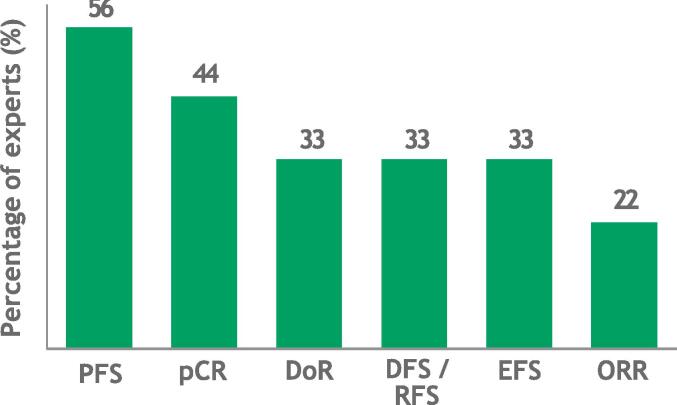
Expert indication of endpoints potentially acceptable for value assessment in oncology.

The group agreed that other oncology-relevant endpoints should be considered when making decisions about access to therapies, with medical oncologists and patient advocates emphasizing the importance of not delaying access to treatment options. Health economists and HTA experts in the group acknowledged the challenges of demonstrating statistically significant OS benefit in early-stage cancers, noting that additional endpoints [17] are increasingly recognized either in their own right or as proxies for OS [18], [19]. Health economists were more likely to accept endpoints for initial value assessment that showed strong correlations with long-term outcomes, although there is currently insufficient evidence for and consensus regarding which alternatives meet these criteria and in which settings (see Fig. 3: results from polling 9 experts on which endpoint(s) are most likely to provide meaningful data on their own in early-stage breast and lung cancers, either as measures of efficacy or predictors of clinical outcomes, depending on the type and stage of cancer).

### 3.2 Principle 2 Continue to build evidence for endpoints that provide earlier indication of treatment efficacy

Emerging additional oncology-relevant endpoints that can detect treatment impact earlier, such as pCR (pathologic complete response, defined as the absence of residual invasive cancer upon evaluation of resected tissue), generally currently require confirmatory longitudinal outcome data. As evidence builds that pCR in specific disease settings and therapeutic classes correlates with other outcomes data (e.g., EFS, DFS and RFS), and/or people with cancer and oncologists confirm it reflects meaningful benefit, pCR may become established as a predictor and/or measure of clinical benefit.

While health economists and regulators in the group preferred OS in the adjuvant setting, they recognized inherent challenges with relying on mature OS data. Patient advocates and oncologists were more amenable to accepting oncology-relevant endpoints such as pCR as measures on their own, without correlating OS, PFS, DFS, or RFS data. The group agreed that PFS, pCR, DoR (duration of response: the length of time a tumour will respond to treatment without growing or metastasizing), DFS, RFS and EFS could provide meaningful efficacy data in early-stage cancers, either as endpoints on their own or in combination, and that the perspectives of people with cancer regarding quality of life (QoL) and tolerability also add value.

pCR is increasingly used as an endpoint in clinical trials and acceptance is growing among regulators [20], [21]. The group explored the role of pCR in early-stage cancers in the neoadjuvant setting as a potential interim oncology-relevant endpoint to support conditional reimbursement approval pending longer-term data. The group indicated that while pCR can provide early data on treatment response, this currently needs to be complemented with longitudinal data to inform regulatory and payer decisions. However, as more trials use pCR as a primary endpoint, stronger evidence will emerge to inform its utility in decision-making and as a predictor of longer-term outcomes.

#### 3.2.1 Principle 3 Develop evidence for the next generation of predictive measures that detect and monitor disease

Advances in disease monitoring, such as through circulating tumour DNA (ctDNA), may provide important early information about treatment response and tumour recurrence. Trials should collect ctDNA data to assess their value as predictors of clinical outcomes.

Precision medicine and research into cancer biomarkers is already yielding results that could transform the management of cancer care in the future. Research into ctDNA shows particular promise. Assessed with a simple blood test (a “liquid biopsy”), ctDNA uses a tumour’s genetic signature to detect disease at a very early stage, allowing for potential early intervention [22].

Research is ongoing across oncology therapy areas into the use of ctDNA to monitor disease and/or predict clinical outcomes, with especially promising studies focusing on metastatic breast cancer [7], [23], [24] and lung cancer [25], [26], [27] as well as early-stage colorectal cancer. The group agreed that further studies are warranted. The group also agreed more broadly that ctDNA has potential as an oncology-relevant measure for value assessment decisions and recommended that ctDNA data collection in clinical trials continue toward that goal. Patient advocates and oncologists noted that uptake of ctDNA monitoring in routine clinical practice would require wider use of liquid biopsies and that monitoring tools would need to be funded adequately and made available to physicians for benefits to be realized in clinical practice.

#### 3.2.2 Principle 4 Use managed entry agreements (MEAs) supported by ongoing evidence collection to help address decision-maker evidence needs

Carefully designed MEAs with planned confirmatory evidence collection can support timely patient access to new therapeutics and help to address evidence uncertainties associated with earlier access for decision makers.

The group highlighted that medicines with a positive value assessment based on predictor endpoints may need additional data to confirm long-term outcomes. As Pauwels et al., wrote “Coverage with evidence agreements can provide answers about uncertainties in real world effectiveness by using patient-relevant outcomes” [28].

The group agreed that, in some countries, carefully designed MEAs with planned confirmatory evidence collection can support timely patient access to new therapeutics and help to address evidence uncertainties. This approach can enable proactive management around uncertainty on the part of the payer related to long-term outcomes and support fast action in clinical practice by working in partnership with the pharmaceutical company to mitigate emerging adverse events not detected during clinical trials. Examples of this approach include the “anwendungsbegleitende Datenerhebung” in Germany [29], “coverage with evidence development” in the US [30] and UK [31], the Italian Medicines Agency (AIFA) registry in Italy [32].

There may also be product- or indication-specific agreements between the manufacturer and payer in which reimbursement is contingent upon pre-specified real-world outcomes [33]. Adjustments to the type of agreement may be needed in countries where citizens pay the majority of medical costs out of pocket.

### 3.3 Principles on value components in oncology

The group agreed that it is useful to consider broader elements of value that early-stage cancer treatments bring to people with cancer, society, and the healthcare system overall. 3 value-related principles were identified.

#### 3.3.1 Principle 5 Routinely use patient reported outcomes (PROs) in value assessments

Data collected from patients via PROs, including QoL, should routinely and consistently be incorporated into value assessments, along with the value components that are already used relating to safety and efficacy.

The group agreed that, in appropriate trials, standard measures of clinical benefit need to be complemented with PROs that assess impact on QoL. The group identified 2 actions that would contribute to this goal:

1. *Improved and more consistent use of QoL data in value assessments.* QoL data are routinely considered in regulators’ assessments as well as in many HTA and payer assessments, but use of these data is not yet universal. Simpler tools are also needed; patient advocate groups often report that existing QoL tools are too complicated or not relevant.
2. *In appropriate trials, utilization of PROs to collect QoL data and support cumulative reporting over time of lower-grade adverse events.* Recalibrating value frameworks to include information on lower grade adverse events that could impact QoL tolerability measures would further support informed decision-making. Patient advocates also encouraged the collection of tolerability data directly via PROs and other mechanisms to get a true understanding of a treatment’s tolerability from an individual’s perspective.

#### 3.3.2 Principle 6 Assess broad economic impact of new medicines

The economic impact of medicines is an essential component of the value assessment and should consider the downstream effect a medicine can have on the amount and associated cost of healthcare resources a patient eventually needs, as well as the socio-economic impact (paid and voluntary work) for patients and those in a caregiving capacity.

Formalized analyses of economic benefit are often limited to costs within the healthcare system [34]. The group discussed additional economic value components suggested by an ISPOR value framework [35], focusing on 2 important factors: a treatment’s socio-economic impact and its impact on healthcare resource utilization overall. The group agreed that the assessment of economic benefit should be widened to include broader societal costs such as the socio-economic impact for people with cancer and those in a caregiving capacity, as well as healthcare expenses that are avoided due to effective earlier treatments. The group agreed that implementing such measurements seemed feasible. Some experts questioned whether this was the role of payers, but there is currently no other established process through which these factors could be assessed.

#### 3.3.3 Principle 7 Consider other value aspects of relevance to patients and society

Insurance value, the value of choice, scientific spillovers, equity of access, and real option value should be considered in value assessments, although they may not all be readily quantifiable and may instead require a more qualitative assessment.

The group reflected on 12 value components considered by an ISPOR special task force [36], highlighting 5 as particularly important.

1) **Insurance value,** as Goring et al. wrote [37], “captures the value to healthy individuals of being protected from the physical and financial burden of a particular illness due to the availability of a new therapy/technology.” Patient advocates valued this component because it provided assurance to people that, should they get sick, treatment options will be available for them.
2) The **value of choice** from having multiple treatment options was vital for patient advocates, with an emphasis on reflecting individual concerns and preferences of people with cancer directly to regulators and payers in order to inform their assessments. (When treatment options have different risk profiles, this additionally brings the **value of hope** into play, as patients will have different risk preferences.).
3) Technological breakthroughs can potentially have **scientific spillover** benefits that enable advances beyond the current product or indication. This should be rewarded to recognize the uncertainties involved in innovation and to provide incentives.
4) **Equity of access** was an important topic as the benefits of therapies are only seen if people with cancer are aware of options, able to access them, and able to stay on treatment [24]. Health inequities are challenging components to measure [38], so the group proposed that innovators should be rewarded for addressing inequities in both drug development and post-approval access designs. Innovators should ensure that their clinical trials recruit participants that reflect the populations impacted by the specific cancer under study, accounting for variation in both genetics and in access to quality care. Increasing the volume of research in this area would give oncologists essential information on dosing and point to ethnic differences in patient response to a given treatment.
5) **Real option value** was identified as important, particularly by patient advocates and oncologists. Real option value is generated when treatments extend the lives and wellbeing of people with cancer so they can benefit from future treatment options and subsequent lines of treatment after their current treatment [39].

## 4 Discussion

Using a modified Delphi process, an expert group reached consensus on 7 principles for defining and assessing the value of cancer therapies. These principles offer a resource to facilitate cross-stakeholder discussion and decision-making, and to support the evolution of existing value frameworks with the goal of improving outcomes for people with cancer.

### 4.1 Oncology Relevant Endpoints

The 4 principles addressing oncology relevant endpoints focus on accelerating the generation of early evidence of treatment effect, while taking appropriate account of considerations regarding long-term effectiveness and safety. The expert group’s consensus on the usefulness of endpoints other than OS reflects and amplifies views that are already being put into practice in some parts of the broader cancer community [17], [18].

A core part of the work that needs to be done to support this trend is the resolution of evidence uncertainties around these alternative endpoints, as this will accelerate access to new medicines that meet the necessary safety, quality, and efficacy thresholds [16]. The group’s view that alternative endpoints could provide meaningful efficacy data in early-stage cancers is consistent with the results of other consensus-building exercises [40]. It is also reflected in practice: clinical trials increasingly use pCR as an oncology-relevant endpoint with promising results [41], [42] and acceptance is growing among regulators [41], [20], [21]. The group also agreed on the importance of developing new predictive measures. The recommendation that ctDNA analysis be explored further is grounded in promising ongoing research into breast and lung cancer [25], [7], [26], [23], [24], [27] and aligns with the emerging consensus around the need to assess this measure’s potential value for people with early-stage solid tumours [43], [44], [40].

Collecting this additional data will address the current shortage of evidence regarding alternative endpoints, which was identified by participating experts as a major barrier to their use. In particular, they highlighted the need for robust evidence that alternative endpoints correlate with longer-term outcomes such as OS or QoL, for specific cancers at specific stages. Likewise, more evidence and deliberation regarding value components will help develop priorities, since not all values can be weighed equally, and some are more challenging to measure than others, from both innovators’ perspective in clinical trials and HTAs’ in terms of value to society and health systems.

While not relevant in all national contexts, carefully designed MEAs have significant potential in some countries for addressing the uncertainty around efficacy endpoints other than OS. In France, for example, the “accès précoce” program enables early access to innovative therapies with the requirement to collect confirmatory real-world evidence on treatments. [45] In the UK, the Cancer Drugs Fund (CDF) has the same intent. The CDF provides funding for early access to new treatments. All patients are registered, allowing the possibility of collection of real-world routine data from electronic patient records and national treatment databases to enable further evaluation [46]. Where benefit is not confirmed by NICE, the CDF stops funding the medicine. This approach is complemented by the Medicines and Healthcare products Regulatory Agency’s increased surveillance and managed access at specialist centers as part of its Early Access to Medicines Scheme (EAMS) [47]. This enables proactive management around uncertainty and fast action within clinical practice working in partnership with the pharmaceutical company to mitigate any emerging adverse events not detected during clinical trials. These programs may serve as useful models for other countries.

### 4.2 Limitations

The principles articulated in this report reflect the perspectives of experts from the cancer communities in 14 countries. While the number of experts who contributed to the consensus meets Delphi process guidelines [48], a larger sample or a sample weighted differently with regard to areas of expertise may not have reached the same consensus. Similarly, the expert perspectives gathered here predominantly represent views from advanced economies. As such, the consensus reached may not be generalizable to the health systems of all countries. Perspectives from beyond the countries represented here are needed in future studies, as care resources, experiences, and outcomes vary widely across the globe.

## 5 Conclusions

### 5.1 Policy recommendations

**Fig. 4:**
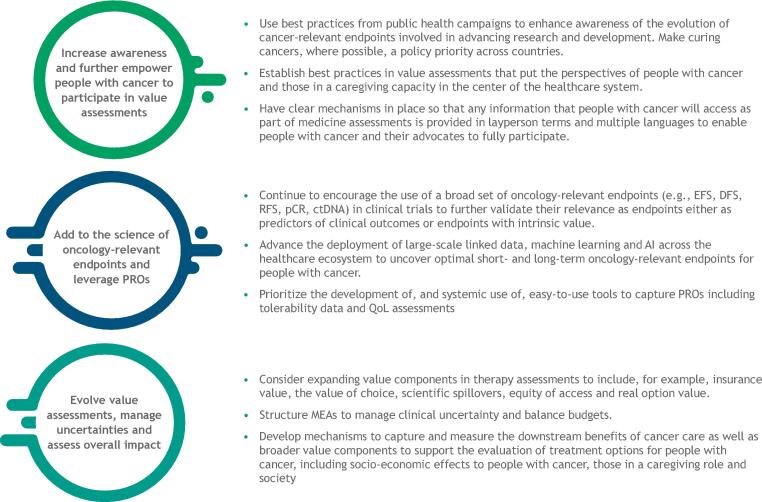
Next steps for evolving value assessment in cancer therapy.

Many countries use value assessment frameworks to inform both clinical and economic decisions about treatment availability and reimbursement. These frameworks assess the essential value components of clinical benefit, economic value, and patient health outcomes alongside a variety of other important measures (see Supplemental Table 2). Examples include the ISPOR value flower [49], value frameworks from the American Society of Clinical Oncology [50], and the National Comprehensive Cancer Network, and the magnitude of clinical benefit scale guideline from the European Society for Medical Oncology [51]. Current value assessments do not always consider the full personal, economic, or societal benefits of early-stage cancer therapies. The principles outlined above can contribute to the evolution of such frameworks by broadening conceptions of value (see Fig. 4).

A key point of agreement among the expert group was that PROs, including QoL, should routinely form part of value assessments to properly reflect the experience of the patient alongside considerations around efficacy and safety. While QoL data are routinely included in many value assessments, such as those of Germany’s Institut für Qualität und Wirtschaftlichkeit im Gesundheitswesen (IQWiG), the US Centers for Medicare & Medicaid Services (CMS) [52] and the UK’s National Institute for Health and Care Excellence (NICE), others, such as private insurers in the U.S., give them less prominence. Consistency on this front is vital. A recent systematic review of the literature determined that incorporating PROs leads to a broad range of care improvements for people with cancer [53]. Efforts are already underway to identify challenges to the collection, analysis, and application of PRO data to inform regulatory and treatment decision making [54], but further attention to this important area is needed.

Assessing the broader economic impact of therapies on society is also an essential step, as narrow analyses that focus solely on their impact within health systems will inevitably obscure the full value of a treatment. Recent research has shown that the return on investment from the societal benefits of new technologies, therapies, and services that extend the prognosis and QoL of people with non-curative cancers can be substantial [55]. A range of metrics for measuring broader socio-economic value components are available in the literature and are feasible to implement [56], [57]. Taking this broader view may provide direct and indirect long-term healthcare savings by reducing the amount of healthcare spending per patient [58], limiting the time they are less able to contribute to the workforce and their communities [5], [59], and easing the emotional stress and financial strain of employment-related issues for their caregivers.

In addition to socio-economic impact, the experts recommended that the cancer community consider 5 additional value components with particular relevance to patients and society: insurance value, value of choice, scientific spillovers, equity of access, and real option value. While no consensus was reached on how to fully capture these components quantitatively, a qualitative assessment could be undertaken for a given cancer indication with input from patient advocates or people with cancer.

### 5.2 Next Steps

Collaboration across the cancer community is essential to make further progress on how value is assessed for early-stage cancer treatments. There are many different roles, perspectives, and areas of expertise across the healthcare ecosystem, but the cancer community shares key common goals: to increase health equity and access, improve the experience for people with cancer from diagnosis through treatment, increase survival rates, and ultimately deliver cures. The value principles outlined in this paper provide a resource for assessing and evaluating innovation toward these goals, with the interests of people with cancer at the core.

The principles offer both a) stimulus and reference points for the evolution of value frameworks used to inform regulatory and reimbursement decision-making, and b) key considerations for the development of medicines. Using these principles as starting points, the cancer community can take specific actions together toward the goal of improving access to new medicines for early-stage cancer.

**AI statement**: The authors made no use of AI for any part of this work.

**Conflict of interest statement:** H.L., M.L., M.G., J.W., and J.G. were funded by AstraZeneca for this research. A.F., Y.K.G, D-C.S. have no conflicts to report, S.B., R.C., P.S, B.S. received funding by AstraZeneca for this research, J.B., R. K., L.G, received honoraria from multiple pharmaceutical companies, T.S. has undertaken consulting/advisory work for multiple pharmaceutical companies, A-M.B. has received honoraria from Roche and AstraZeneca, and participates in patient councils and advisory boards with proceeds from those going to Lung Cancer Europe (LuCE); J.C. has (1) undertaken consulting/advisory work for: Roche, AstraZeneca, Seattle Genetics, Daiichi Sankyo,Lilly, Merck Sharp&Dohme, Leuko, Bioasis, Clovis Oncology, Boehringer Ingelheim, Ellipses, Hibercell, BioInvent, Gemoab, Gilead, Menarini, Zymeworks, Reveal Genomics, Scorpion Therapeutics, Expres2ion Biotechnologies, Jazz Pharmaceuticals, Abbvie, (2) received honoraria from Roche, Novartis, Eisai, Pfizer, Lilly, Merck Sharp&Dohme, Daiichi Sankyo, Astrazeneca, Gilead, Steamline Therapeutics, (3) received esearch funding to the Institution from Roche, Ariad pharmaceuticals, AstraZeneca, Baxalta GMBH/Servier Affaires, Bayer healthcare, Eisai, F. Hoffman-La Roche, Guardanth health, Merck Sharp&Dohme, Pfizer, Piqur Therapeutics,Iqvia, Queen Mary University of London, and (4) holds stock in MAJ3 and Capital; G.C. has (1) undertaken consulting advisory work for BMS, Roche, Pfizer, Novartis, Lilly, Astra Zeneca, Daichii Sankyo, Merck, Seagen, Ellipsis, Gilead, and (2) received honoraria from Lilly, Pfizer, Relay; G.L. has undertaken consulting advisory work for Sandoz; G1 Therapeutics; Partners Healthcare; BeyondSpring; ER Squibb; MSD; Jazz Pharm; TEVA; Seattle Genetics; Fresenius Kabi; and Samsung (all outside the submitted work); L.P. has received consultancies funding by pharmaceutical companies and owns stocks / options in two of them; G.V. has (1) undertaken consulting/advisory work for AstraZeneca, Roche, Novartis, Pfizer, Seagen, Eisai, Sanofi and (2) received honoraria from Pfizer, Novartis, Eisai; Z.P-W has received grant funding from and/or participated in an advisory board in the last 24 months from the following pharmaceutical companies: AbbVie, Adaptive, Amgen, Autolus, Astellas, AstraZeneca, Bristol-Myers Squibb, CanCell Therapeutics, Daiichi-Sankyo, Gilead, Glycostem, Incyte, INO Therapeutics, Janssen, Jazz, Kura Oncology, Kyowa Kirin, Novartis, Otsuka, Pleco Therapeutics, Pfizer, Roche, Seagen, Servier, Sobi, Takeda and Zambon.

## Funding information

This research was funded by AstraZeneca.

## CRediT authorship contribution statement

**Myrto Lee:** Writing – review & editing, Supervision, Project administration, Methodology, Investigation, Funding acquisition, Conceptualization. **Hugo Larose:** Writing – review & editing, Writing – original draft, Project administration, Methodology, Investigation, Formal analysis, Data curation, Conceptualization, Methodology. **Martin Gräbeldinger:** Writing – review & editing, Writing – original draft, Data curation. **Jon Williams:** Supervision, Project administration, Methodology, Investigation, Funding acquisition, Conceptualization, Methodology. **Anne-Marie Baird:** Writing – review & editing, Validation, Investigation. **Susan Brown:** Writing – review & editing, Validation, Investigation. **Johannes Bruns:** Writing – review & editing, Validation, Investigation. **Russell Clark:** Writing – review & editing, Validation, Investigation. **Javier Cortes:** Writing – review & editing, Validation, Investigation. **Giuseppe Curigliano:** Writing – review & editing, Validation, Investigation. **Andrea Ferris:** Writing – review & editing, Validation, Investigation. **Louis P. Garrison:** Writing – review & editing, Validation, Investigation. **Y.K. Gupta:** Writing – review & editing, Validation, Investigation. **Ravindran Kanesvaran:** Writing – review & editing, Validation, Investigation. **Gary Lyman:** Writing – review & editing, Validation, Investigation. **Luca Pani:** Writing – review & editing, Validation, Investigation. **Zack Pemberton-Whiteley:** Writing – review & editing, Validation, Investigation. **Tomas Salmonson:** Writing – review & editing, Validation, Investigation. **Peter Sawicki:** Writing – review & editing, Validation, Investigation. **Barry Stein:** Writing – review & editing, Validation, Investigation. **Dong-Churl Suh:** Writing – review & editing, Validation, Investigation. **Galina Velikova:** Writing – review & editing, Validation, Investigation. **Jens Grueger:** Methodology, Writing – review & editing, Supervision, Project administration, Methodology, Investigation, Funding acquisition, Conceptualization.

## Declaration of competing interest

The authors declare that they have no known competing financial interests or personal relationships that could have appeared to influence the work reported in this paper.
