# Supplementary material for: The evolving value assessment of cancer therapies: Results from a modified Delphi study
Source: Health Policy Open. 2024 Mar 1;6:100116. doi: 10.1016/j.hpopen.2024.100116 (PMC10924144; doi:10.1016/j.hpopen.2024.100116)
Supplement: Supplementary data 1 [file mmc1.docx]

**Supplemental material: expert panelists and interviewees who participated.**

| Type | **Name** | **Role** | **Expertise / Country** |
| --- | --- | --- | --- |
| **Expert Panelists** | Keith Abrams, PhD | Professor of Statistics & Data Science, University of Warwick | Health Economist  UK |
|  | Susan Brown, RN | Senior Director, Education & Patient Support,​ Susan G. Komen Foundation | Patient Advocate  USA |
|  | Johannes Bruns, MD | General Secretary of Deutsche Krebsgesellschaft (German Cancer Society) | Oncologist  Germany |
|  | John Carpten, MD | Director, National Cancer Advisory Board; Professor and Chair of Translational Genomics and Director of the Institute of Translational Genomics, Royce and Mary Trotter Chair in Cancer Research, Keck School of Medicine, University of Southern California | Oncologist  USA |
|  | Russell Clark | President of Cancer Technology Applications. Senior Vice President Business Development at Spesana, Inc | Health Economist  USA |
|  | Javier Cortes, MD, PhD | Head at International Breast Cancer Center | Oncologist  Spain |
|  | Giuseppe Curigliano, MD, PhD | Professor of Medical oncology, Milan University; Chair of the ESMO guidelines committee | Oncologist  Italy |
|  | Andrea Ferris | President and CEO of LUNGevity | Patient Advocate  USA |
|  | Louis P. Garrison, PhD | Emeritius Professor at University of Washington, Former President of ISPOR (2016-17) | Health Economist  USA |
|  | Gary Lyman, MD, PhD | Professor of Public Health Sciences and Clinical Research Fred Hutchinson Cancer Research Center | Oncologist  USA |
|  | Luca Pani, MD | Professor of Psychiatry at University of Miami and Professor of Pharmacology at Università di Modena e Reggio Emilia | Health Technology Assessment, Regulator  Italy |
|  | Zack Pemberton-Whiteley | CEO of Leukaemia Care | Patient Advocate  UK |
|  | Tomas Salmonson, MD, PhD | Partner at Consilium Salmonson & Hemmings. Former Chair of Committee for Medicinal Products for Human at EMA (2012-18) | Regulator  Sweden |
|  | Peter Sawicki, MD, PhD | University of Cologne, Partner at Dr. Bettina Weihe und Prof. Peter T. Sawicki. Former Head of IQWiG (2004-10) | Health Technology Assessment  Germany |
|  | Richard Vines | Director, Rare Cancers Australia | Patient Advocate  Australia |
| **Expert Interviewees** | Anne-Marie Baird, PhD | President, Lung Cancer Europe | Patient Advocate  Ireland |
|  | Y. K. Gupta, MD | President, All India Institute of Medical Science Bhopal | Oncologist  India |
|  | Ataru Igarashi, PhD | Associate Professor, Unit of Public Health and Preventative Medicine, Yokohama City University School of Medicine | Health Economist  Japan |
|  | Ravindran Kanesvaran, MD | Deputy Chair and Senior Consultant; Associate Professor Division of Medical Oncology, National Cancer Centre Singapore | Oncologist  Singapore |
|  | Kun Zhao, MD, PhD | Professor, Division of Health Policy Evaluation and Technology Assessment; China National Health Development Research Center | Health Economist  China |
|  | Barry Stein | President - CEO, Cancer Colorectal Canada | Patient Advocate  Canada |
|  | Dong-Churl Suh, PhD | Director, Institute of Pharmaceutical Economics and Policy, Chung-Ang University | Health Technology Assessment  South Korea |
|  | Galina Velikova, MD | Professor Psychosocial and Medical Oncology, University of Leeds | Oncologist  UK |

**Off-line survey:**

**Cancer community consensus: the evolving definition of value of oncology medicines**

**Part 0 of 2: Your background & thoughts on project outcomes**

**0.A What is your name?**

**0.B In which of the following bodies do you have direct experience working?
*Please select multiple if applicable.***

Payer

Health Technology Assessment Body

Regulator

Patient Advocacy Group

Medical Society

Other

**0.C In which countries/regions do you have experience?
*Please select multiple if applicable.***

Australia

Canada

China

EU

France

Germany

Italy

Japan

South Korea

Spain

UK

US

Other

None of the above

# Part 1 of 2: Deep-dive questions into the four value dimensions


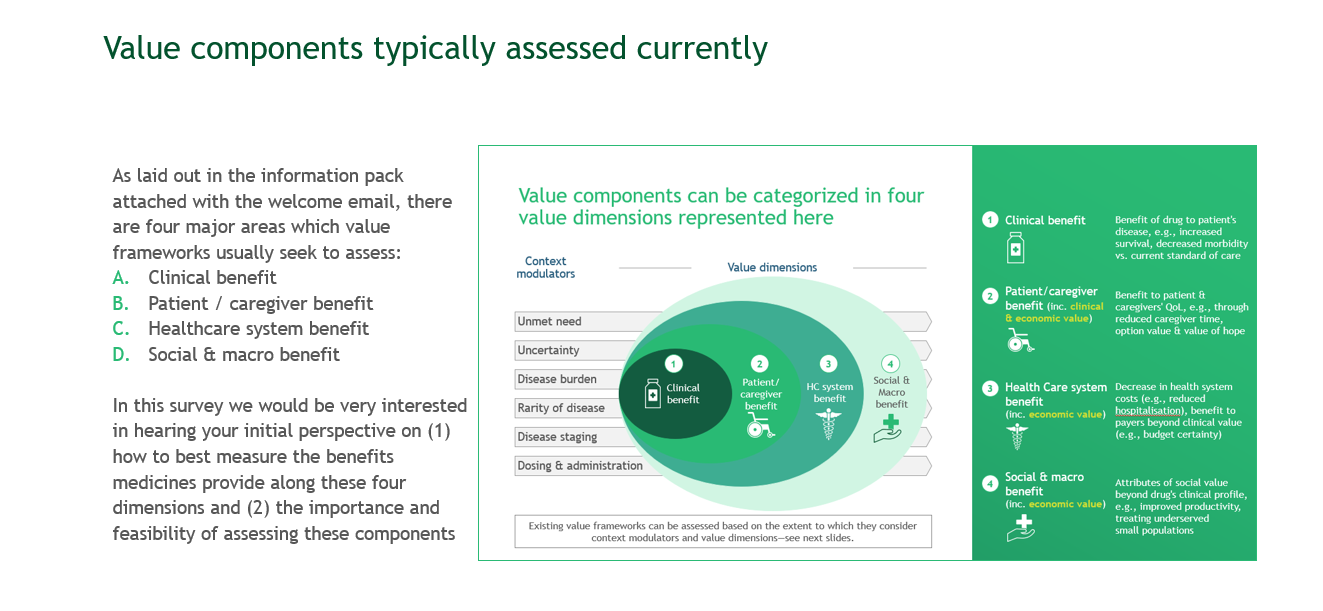


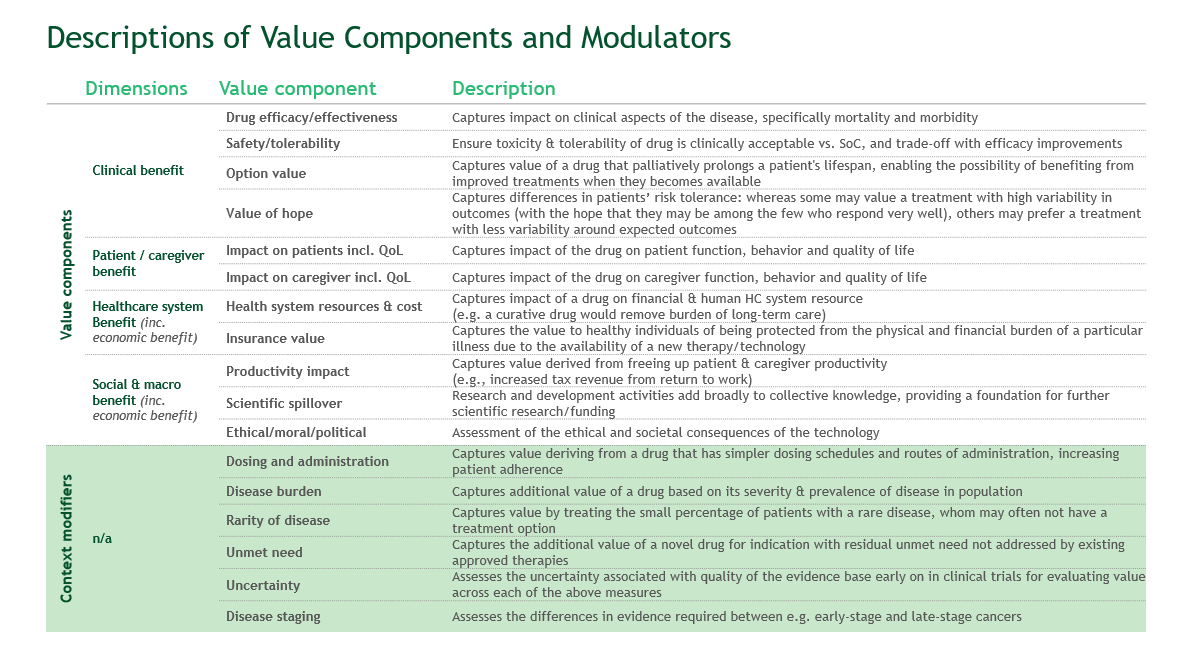


**1.A To what extent do you think that regulators and payers value the following four dimensions currently?**

***Please choose one for each dimension and explain your assessment***

**Clinical benefit**

Highly valued

Somewhat valued

Not valued

**Benefit to patient and/or caregivers**

Highly valued

Somewhat valued

Not valued

**Benefit to the healthcare system**

Highly valued

Somewhat valued

Not valued

**Social/macro benefit**

Highly valued

Somewhat valued

Not valued

# Deep-dive on clinical endpoints


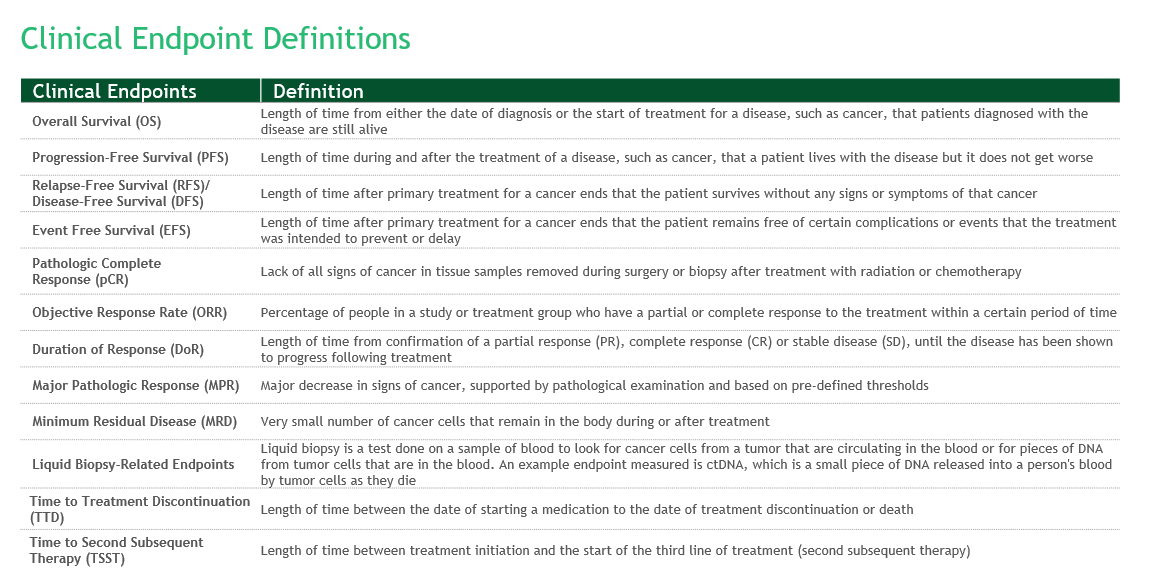
***We are interested in understanding your view on suitability (i.e. ability to evaluate both safety and drug efficacy while providing timely patient access) of each clinical endpoint to be considered for early-stage (Stage I/II, non-metastatic disease) and late-stage (Stage III/IV, locally advanced or metastatic disease) cancer trials. Specifically we are interested in breast & lung cancers as examples where there currently is most literature published, though we are also interested in hearing your thoughts on other solid cancers and haematological tumours, where non-OS endpoints may be more commonly accepted.***

**1.G [Breast cancer] How suitable do you think the following endpoints are in breast cancer?**

**Overall Survival (OS) - early-stage**

Not suitable

Suitable in select circumstances

Suitable in most circumstances

Always suitable

**Overall Survival (OS) - late-stage**

Not suitable

Suitable in select circumstances

Suitable in most circumstances

Always suitable

**Progression-Free Survial (PFS) - early-stage**

Not suitable

Suitable in select circumstances

Suitable in most circumstances

Always suitable

**Progression-Free Survial (PFS) - late-stage**

Not suitable

Suitable in select circumstances

Suitable in most circumstances

Always suitable

**Relapse Free Survival/Disease Free Surival (RFS/DFS) - early-stage**

Not suitable

Suitable in select circumstances

Suitable in most circumstances

Always suitable

**Relapse Free Survival/Disease Free Surival (RFS/DFS) - late-stage**

Not suitable

Suitable in select circumstances

Suitable in most circumstances

Always suitable

**Event-Free Survival (EFS) - early-stage**

Not suitable

Suitable in select circumstances

Suitable in most circumstances

Always suitable

**Event-Free Survival (EFS) - late-stage**

Not suitable

Suitable in select circumstances

Suitable in most circumstances

Always suitable

**Pathologic complete response (pCR) – early-stage**

Not suitable

Suitable in select circumstances

Suitable in most circumstances

Always suitable

**Pathologic complete response (pCR) – late-stage**

Not suitable

Suitable in select circumstances

Suitable in most circumstances

Always suitable

**Objective Reponse Rate (ORR) – early-stage**

Not suitable

Suitable in select circumstances

Suitable in most circumstances

Always suitable

**Objective Reponse Rate (ORR) – late-stage**

Not suitable

Suitable in select circumstances

Suitable in most circumstances

Always suitable

**Duration of Response (DoR) – early-stage**

Not suitable

Suitable in select circumstances

Suitable in most circumstances

Always suitable

**Duration of Response (DoR) – late-stage**

Not suitable

Suitable in select circumstances

Suitable in most circumstances

Always suitable

**Major Pathologivc Reponse (MPR) – early-stage**

Not suitable

Suitable in select circumstances

Suitable in most circumstances

Always suitable

**Major Pathologic Reponse (MPR) – late-stage**

Not suitable

Suitable in select circumstances

Suitable in most circumstances

Always suitable

**Minimum Residual Disease (MRD) – early-stage**

Not suitable

Suitable in select circumstances

Suitable in most circumstances

Always suitable

**Minimum Residual Disease (MRD) – late-stage**

Not suitable

Suitable in select circumstances

Suitable in most circumstances

Always suitable

**Liquid Biopsy-Related Endpoints (LBRE) – early-stage**

Not suitable

Suitable in select circumstances

Suitable in most circumstances

Always suitable

**Liquid Biopsy-Related Endpoints (LBRE) – late-stage**

Not suitable

Suitable in select circumstances

Suitable in most circumstances

Always suitable

**Time to Treatment Discontinuation (TTD) ) – early-stage**

Not suitable

Suitable in select circumstances

Suitable in most circumstances

Always suitable

**Time to Treatment Discontinuation (TTD) ) – late-stage**

Not suitable

Suitable in select circumstances

Suitable in most circumstances

Always suitable

**Time to Second Subsequent Therapy (TSST) – early-stage**

Not suitable

Suitable in select circumstances

Suitable in most circumstances

Always suitable

**Time to Second Subsequent Therapy (TSST) – late-stage**

Not suitable

Suitable in select circumstances

Suitable in most circumstances

Always suitable

**1.H [Lung cancer] How suitable do you think the following endpoints are in lung cancer?**

**Overall Survival (OS) - early-stage**

Not suitable

Suitable in select circumstances

Suitable in most circumstances

Always suitable

**Overall Survival (OS) - late-stage**

Not suitable

Suitable in select circumstances

Suitable in most circumstances

Always suitable

**Progression-Free Survial (PFS) - early-stage**

Not suitable

Suitable in select circumstances

Suitable in most circumstances

Always suitable

**Progression-Free Survial (PFS) - late-stage**

Not suitable

Suitable in select circumstances

Suitable in most circumstances

Always suitable

**Relapse Free Survival/Disease Free Surival (RFS/DFS) - early-stage**

Not suitable

Suitable in select circumstances

Suitable in most circumstances

Always suitable

**Relapse Free Survival/Disease Free Surival (RFS/DFS) - late-stage**

Not suitable

Suitable in select circumstances

Suitable in most circumstances

Always suitable

**Event-Free Survival (EFS) - early-stage**

Not suitable

Suitable in select circumstances

Suitable in most circumstances

Always suitable

**Event-Free Survival (EFS) - late-stage**

Not suitable

Suitable in select circumstances

Suitable in most circumstances

Always suitable

**Pathologic complete response (pCR) – early-stage**

Not suitable

Suitable in select circumstances

Suitable in most circumstances

Always suitable

**Pathologic complete response (pCR) – late-stage**

Not suitable

Suitable in select circumstances

Suitable in most circumstances

Always suitable

**Objective Reponse Rate (ORR) – early-stage**

Not suitable

Suitable in select circumstances

Suitable in most circumstances

Always suitable

**Objective Reponse Rate (ORR) – late-stage**

Not suitable

Suitable in select circumstances

Suitable in most circumstances

Always suitable

**Duration of Response (DoR) – early-stage**

Not suitable

Suitable in select circumstances

Suitable in most circumstances

Always suitable

**Duration of Response (DoR) – late-stage**

Not suitable

Suitable in select circumstances

Suitable in most circumstances

Always suitable

**Major Pathologivc Reponse (MPR) – early-stage**

Not suitable

Suitable in select circumstances

Suitable in most circumstances

Always suitable

**Major Pathologic Reponse (MPR) – late-stage**

Not suitable

Suitable in select circumstances

Suitable in most circumstances

Always suitable

**Minimum Residual Disease (MRD) – early-stage**

Not suitable

Suitable in select circumstances

Suitable in most circumstances

Always suitable

**Minimum Residual Disease (MRD) – late-stage**

Not suitable

Suitable in select circumstances

Suitable in most circumstances

Always suitable

**Liquid Biopsy-Related Endpoints (LBRE) – early-stage**

Not suitable

Suitable in select circumstances

Suitable in most circumstances

Always suitable

**Liquid Biopsy-Related Endpoints (LBRE) – late-stage**

Not suitable

Suitable in select circumstances

Suitable in most circumstances

Always suitable

**Time to Treatment Discontinuation (TTD) ) – early-stage**

Not suitable

Suitable in select circumstances

Suitable in most circumstances

Always suitable

**Time to Treatment Discontinuation (TTD) ) – late-stage**

Not suitable

Suitable in select circumstances

Suitable in most circumstances

Always suitable

**Time to Second Subsequent Therapy (TSST) – early-stage**

Not suitable

Suitable in select circumstances

Suitable in most circumstances

Always suitable

**Time to Second Subsequent Therapy (TSST) – late-stage**

Not suitable

Suitable in select circumstances

Suitable in most circumstances

Always suitable

**1.L How do you see these clinical endpoints evolving in future? Are you aware of any new emerging endpoints? What opportunities do these endpoints offer the cancer community?**

# Part 2 of 2: Importance & feasibility of measuring value components

**While there is agreement in the cancer community on the importance of measuring the clinical efficacy and safety of medicines, as well as healthcare resource use and cost, a number of additional value components have been proposed (eg. the**[***ISPOR Value Flower***](https://www.ispor.org/publications/journals/value-outcomes-spotlight/vos-archives/issue/view/navigating-the-changing-heor-publishing-landscape/novel-elements-of-the-value-flower-fake-or-truly-novel)**). In Part 2 of this survey we would be very interested in hearing your perspective on the importance & feasibility of assessing value components.

Unless otherwise specified, for each of the following questions, we are interested in your perspective on value assessment of a new oncology medicine.**

**2.B For each of the following value components, what do you think is the *importance of considering*them in value assessments for *early-stage (Stage I/II, non-metastatic disease) cancers?* *Please choose one of the following for each value component***

**Drug efficacy/effectiveness**

Not important

Important in a few circumstances

Important in most circumstances

Always important

**Safety/tolerability**

Not important

Important in a few circumstances

Important in most circumstances

Always important

**Option value**

Not important

Important in a few circumstances

Important in most circumstances

Always important

**Value of hope**

Not important

Important in a few circumstances

Important in most circumstances

Always important

**Impact on patients (inc. QoL)**

Not important

Important in a few circumstances

Important in most circumstances

Always important

**Impact on caregivers (inc. QoL)**

Not important

Important in a few circumstances

Important in most circumstances

Always important

**Health system resources & costs**

Not important

Important in a few circumstances

Important in most circumstances

Always important

**Insurance value**

Not important

Important in a few circumstances

Important in most circumstances

Always important

**Productivity impact**

Not important

Important in a few circumstances

Important in most circumstances

Always important

**Scientific Spillover**

Not important

Important in a few circumstances

Important in most circumstances

Always important

**Ethical / moral / political**

Not important

Important in a few circumstances

Important in most circumstances

Always important

**2.C For each of the following value components, what do you think is the importance of including them in value assessments for late-stage (Stage III/IV, locally advanced or metastatic disease) cancers?**

**Drug efficacy/effectiveness**

Not important

Important in a few circumstances

Important in most circumstances

Always important

**Safety/tolerability**

Not important

Important in a few circumstances

Important in most circumstances

Always important

**Option value**

Not important

Important in a few circumstances

Important in most circumstances

Always important

**Value of hope**

Not important

Important in a few circumstances

Important in most circumstances

Always important

**Impact on patients (inc. QoL)**

Not important

Important in a few circumstances

Important in most circumstances

Always important

**Impact on caregivers (inc. QoL)**

Not important

Important in a few circumstances

Important in most circumstances

Always important

**Health system resources & costs**

Not important

Important in a few circumstances

Important in most circumstances

Always important

**Insurance value**

Not important

Important in a few circumstances

Important in most circumstances

Always important

**Productivity impact**

Not important

Important in a few circumstances

Important in most circumstances

Always important

**Scientific Spillover**

Not important

Important in a few circumstances

Important in most circumstances

Always important

**Ethical / moral / political**

Not important

Important in a few circumstances

Important in most circumstances

Always important

**2.E For each of the following clinical & economic value components, how *feasible*do you think it is to provide robust evidence for value assessment of a new therapy for early-stage (Stage I/II, non-metastatic disease) cancers?**

**Drug efficacy/effectiveness**

Not feasible

Sometimes feasible

Often feasible

Always feasible

**Safety/tolerability**

Not feasible

Sometimes feasible

Often feasible

Always feasible

**Option value**

Not feasible

Sometimes feasible

Often feasible

Always feasible

**Value of hope**

Not feasible

Sometimes feasible

Often feasible

Always feasible

**Impact on patients (inc. QoL)**

Not feasible

Sometimes feasible

Often feasible

Always feasible

**Impact on caregivers (inc. QoL)**

Not feasible

Sometimes feasible

Often feasible

Always feasible

**Health system resources & costs**

Not feasible

Sometimes feasible

Often feasible

Always feasible

**Insurance value**

Not feasible

Sometimes feasible

Often feasible

Always feasible

**Productivity impact**

Not feasible

Sometimes feasible

Often feasible

Always feasible

**Scientific Spillover**

Not feasible

Sometimes feasible

Often feasible

Always feasible

**Ethical / moral / political**

Not feasible

Sometimes feasible

Often feasible

Always feasible

**2.G For each of the following clinical & economic value components, how *feasible*do you think it is of providing robust evidence for value assessment of a new therapy for late-stage (Stage III/IV, locally advanced or metastatic disease) cancers.**

**Drug efficacy/effectiveness**

Not feasible

Sometimes feasible

Often feasible

Always feasible

**Safety/tolerability**

Not feasible

Sometimes feasible

Often feasible

Always feasible

**Option value**

Not feasible

Sometimes feasible

Often feasible

Always feasible

**Value of hope**

Not feasible

Sometimes feasible

Often feasible

Always feasible

**Impact on patients (inc. QoL)**

Not feasible

Sometimes feasible

Often feasible

Always feasible

**Impact on caregivers (inc. QoL)**

Not feasible

Sometimes feasible

Often feasible

Always feasible

**Health system resources & costs**

Not feasible

Sometimes feasible

Often feasible

Always feasible

**Insurance value**

Not feasible

Sometimes feasible

Often feasible

Always feasible

**Productivity impact**

Not feasible

Sometimes feasible

Often feasible

Always feasible

**Scientific Spillover**

Not feasible

Sometimes feasible

Often feasible

Always feasible

**Ethical / moral / political**

Not feasible

Sometimes feasible

Often feasible

Always feasible

**2.K To what extent do any of the following considerations influence the value assessment? For example would a therapy that is easier to use in a high unmet need area be considered more valuable than another therapy with comparable efficacy and safety? *Please choose one of the following for each context modulator & include your rationale in the space provided***

**Dosing and administration (with potential impact on adherence)**

No impact

Low impact

High impact

**Disease burden (high prevalence)**

No impact

Low impact

High impact

**Rarity of disease**

No impact

Low impact

High impact

**Unmet need (lack of appropriate treatment alternatives)**

No impact

Low impact

High impact

**Uncertainty (associated with smaller evidence base early on in clinical trials)**

No impact

Low impact

High impact

**Disease staging (early vs late-stage cancer)**

No impact

Low impact

High impact
