# Supplementary material for: The evolving value assessment of cancer therapies: Results from a modified Delphi study
Source: Health Policy Open. 2024 Mar 1;6:100116. doi: 10.1016/j.hpopen.2024.100116 (PMC10924144; doi:10.1016/j.hpopen.2024.100116)
Supplement: Supplementary data 2 [file mmc2.docx]

**Supplemental table 1: Oncology-relevant endpoints.** Unless otherwise noted, definitions are from the National Cancer Institute (NCI) Dictionary of Cancer Terms. Endpoints are listed alphabetically

| **Additional oncology-relevant endpoints** | Additional oncology-relevant endpoints (beyond OS) that are often used in early-stage cancer could include those listed below; the following are referenced in this paper: DFS, RFS, DoR EFS, pCR, ORR. They should be informed by the needs of all relevant stakeholders including payers, regulators, patients and oncologists. |
| --- | --- |
| **Disease-Free Survival (DFS) / Relapse-Free Survival (RFS)** | Length of time after primary treatment for a cancer ends that the patient survives without any signs or symptoms of that cancer. Also called relapse-free survival. |
| **Duration of Response (DoR)** | Length of time from randomization to disease progression or death in patients who achieve complete or partial response; measures how long a patient will respond to treatment without tumor growth or metastasis. |
| **Event-Free Survival (EFS)** | Length of time after primary treatment for a cancer ends that the patient remains free of certain complications or events that the treatment was intended to prevent or delay. These events may include the return of the cancer or the onset of certain symptoms, such as bone pain from cancer that has spread to the bone. |
| **Liquid Biopsy (LB)-related endpoints** | LBs are a broad concept that encompasses the analysis of circulating nucleic acids, tumor cells or exosomes as a tool to molecularly profile tumors to guide clinical decision making |
| **Major Pathologic Response (MPR)** | The reduction to 10% or less residual tumour after therapy. |
| **Minimum Residual Disease (MRD)** | A reduction to sub-microscopic levels of residual tumour cells. |
| **Pathologic Complete Response (pCR)** | The lack of all signs of cancer in tissue samples removed during surgery or biopsy after treatment such as that with radiation or chemotherapy. |
| **Progression-Free Survival (PFS)** | Length of time during and after the treatment of a disease, such as cancer, that a patient lives with the disease but it does not get worse. |
| **Overall (or Objective) Response Rate (ORR)** | Percentage of people in a study or treatment group who have a partial or complete response to the treatment within a certain period of time. A partial response is a decrease in the size of a tumor or in the amount of cancer in the body, and a complete response is the disappearance of all signs of cancer in the body. |
| **Overall Survival (OS)** | The length of time from either the date of diagnosis or the start of treatment for a disease, such as cancer, that patients diagnosed with the disease are still alive. |
| **Time to Second Subsequent Therapy (TSST)** | The length of time between treatment initiation and the start of the third line of treatment (second subsequent therapy). |
| **Time to Treatment Discontinuation (TTD)** | The length of time from the start of therapy to the time of treatment discontinuation for any reason. |

**Supplemental table 2: Value components in Oncology.** Components are listed alphabetically

| **Equity of access** | Promotes equal access for equal need. |
| --- | --- |
| **Economic value** | Considers value for money. Includes measurement of direct and indirect costs to the healthcare system, individuals and society. |
| **Impact on caregivers** | Impact on a caregiver such as on their time and QoL. |
| **Impact on patients** | Impact on a patient, for example, on patient function and QoL. |
| **Indirect costs** | These may include loss of income and other expenses for working-age patients, and, on aggregate, the financial burden to society due to reduced or lost productivity across the workforce. |
| **Insurance value** | Value to healthy individuals of being protected from the physical and financial burden of illness due to the availability of a new medicine or technology |
| **Patient Reported Outcomes (PROs)** | Report of the status of a patient’s health condition or QoL measures that come directly from the patient. |
| **Quality-Adjusted Life Years (QALY)** | A measure of the state of health of a person or group in which the benefits, in terms of length of life, are adjusted to reflect the quality of life |
| **Quality of Life (QoL)** | An individual’s perception of their position in life in the context of the culture and value systems in which they live and in relation to their goals, expectations, standards and concerns^16^. Health-related quality of life is a combination of a person’s physical, mental and social well-being; not merely the absence of disease |
| **Real option value** | Value of a therapy that helps maintain a person’s health status, enabling the possibility of benefitting from future medical treatments when they become available^24^. |
| **Safety** | The safety of a medical product concerns the medical risk to the patient, usually assessed in a clinical trial by laboratory tests (including clinical chemistry and hematology), vital signs, clinical adverse events (diseases, signs and symptoms) and other special safety tests |
| **Tolerability** | Represents the degree to which overt adverse effects can be tolerated by a patient |
| **Socio-economic impact** | This includes the effect a treatment has on a patient or caregiver’s ability to engage in paid and voluntary work. |
| **Value of choice** | Gives weight to the importance of treatment options, and the more the better. |
| **Value of hope** | Differences in patients’ risk tolerance: some may value a treatment with high variability in outcomes, with the hope that they may be fortunate and respond very well. |
